# Excessive daytime sleepiness in myotonic dystrophy: a narrative review

**DOI:** 10.3389/fneur.2024.1389949

**Published:** 2024-07-01

**Authors:** Domeniko Hoxhaj, Alessia Pascazio, Michelangelo Maestri, Giulia Ricci, Monica Fabbrini, Francesca Buracchi Torresi, Gabriele Siciliano, Enrica Bonanni

**Affiliations:** Neurology Unit, Department of Clinical and Experimental Medicine, University of Pisa, Pisa, Italy

**Keywords:** neuromuscolar disorders, sleep disorder, myotonic distrophies, sleepeness, genetic disorder

## Abstract

**Introduction:**

Excessive daytime sleepiness (EDS) is a common and debilitating symptom in both forms of myotonic dystrophy (DM), significantly impacting patients’ quality of life. The review focuses on the purpose of examining the current understanding of EDS in these conditions, the difficulty in correctly accessing it, the recent findings related to its etiology and prevalence, and a summary of potential therapeutic implications.

**Methods:**

We conducted a comprehensive search through PubMed, selecting studies that provided significant insights into the mechanisms, prevalence, and management of EDS in DM1 and DM2.

**Results and discussion:**

EDS is highly prevalent in both DM1 and DM2. Polysomnographic studies have revealed prominent dysregulation of REM sleep in DM1, suggesting a possible narcoleptic-like phenotype and alterations in NREM sleep that contributes to daytime sleepiness. Other factors have been proposed to explain EDS in DM1, including dysregulation of the sleep-wake circadian rhythm through nocturnal actigraphy analysis. The central origin of EDS is increasingly delineated supported by serotonin and orexin pathways dysfunction, and recent neuroradiological findings showing that in DM1 hippocampus volume was positively correlated with self-reported fatigue and somnolence. Sleep-disordered breathing and respiratory dysfunctions are prevalent in DM, their direct correlation with EDS remains complex and inconclusive, but respiratory evaluation should be recommended if obstructive sleep apneas or respiratory muscle dysfunctions are suspected. Drug interventions, such as modafinil and mexiletine, have shown promise in managing excessive daytime sleepiness and reducing myotonia without significant cardiac conduction effects. Enhancing EDS management in myotonic dystrophy is key to improving overall patient well-being.

## Introduction

Myotonic dystrophy (DM) is a group of autosomal dominant genetic disorders that encompasses myotonic dystrophy type 1 (DM1) and myotonic dystrophy type 2 (DM2). These two forms of the disease differ at the genetic level, with DM1 stemming from an expanded CTG triplet in the DMPK gene on chromosome 19 and DM2 resulting from an expansion of a CCTG tetramer in the CNBP gene, also known as ZNF9, in chromosome 3 (1, 2). However, both disorders share significant pathogenetic features that lead to overlapping clinical manifestations. In both forms the expanded DNA is transcribed into expanded RNA, which disrupts cellular processes governing gene expression. DMPK and CNBP are expressed in various tissue types, including skeletal and smooth muscle, cardiac, central nervous system (CNS), and eye, giving rise to a wide range of multi-organ involvement, such as muscle atrophy, weakness, myotonia, cardiac arrhythmias, posterior cataracts, diabetes mellitus, cognitive impairments, risk of infertility, and psychiatric disturbances (3).

Sleep disorders play a pivotal role in affecting the daytime functioning of patients with DM, with excessive daytime sleepiness (EDS) being a prevalent and debilitating symptom in both forms (4). The exact nature of EDS, its origins, and methods to assess it are still not completely clear. Several studies support the hypothesis of a central nervous system dysfunction of sleep regulation. Moreover, some studies suggest that, in some patients with DM1, EDS may also be related to a sleep fragmentation induced by a sleep-related breathing disorder (SDB) such as central or obstructive apnea, or it may be secondary to nocturnal hypoxemia and diurnal hypercapnia (5).

This narrative review aims at discussing sleep disorders in DM1 and DM2, focusing on EDS. In doing so, the latest insights into the likely pathophysiological mechanisms behind it will be discussed, along with possible current therapeutic approaches, without forgetting the differences between the two forms of DM: DM1 and DM2.

## Methods

To perform this review PubMed was searched until October 2023 for “myotonic dystrophy AND sleep disorders,” “myotonic dystrophy AND sleep,” “myotonic dystrophy AND sleepiness,” “myotonic dystrophy AND excessive daytime sleepiness.” We selected both clinical studies and reviews assessing these topics. In total we included 30 papers in the review (see Bibliography). Additional research was addressed when describing DM1 and DM2 clinical features.

## Sleep disorders as clinical manifestation of DM1: focus on EDS

DM1 exhibits a prevalence ranging from 5 to 20 cases per 100,000 individuals (6), making it one of the most common muscular dystrophies among adults. The primary muscles affected are those of the face and distal limbs, with proximal muscles often becoming involved in later stages of the disease. Patients may also experience symptoms like dysarthria, dysphagia, and respiratory muscle weakness (7). The onset of clinical symptoms varies widely and is primarily determined by genetic factors. The severity of DM1 correlates with the number of expanded CTG triplet and it could be classified (8) in:

- Congenital DM1 is the most severe form in infant and it presents with marked hypotonia rather than myotonia. As a consequence, dysphagia, which would lead to failure to thrive, requires a nasogastric or gastric feeding tube. In addition to that, one of the most important issues is the global developmental delay, which would help in the differential to other severe congenital myopathies (9, 10).
- Childhood DM1 typically manifests before the age of 10, often accompanied by cognitive, behavioral and mood disturbances. Muscle symptoms and physical disability develop over time (11).
- Classic DM1 usually becomes symptomatic between the second and fourth decade of life. Major issues include respiratory muscle weakness, myotonia, cataracts, cardiac arrhythmias, and EDS. Life expectancy is reduced (12).
- Mild DM1 typically presents after the age of 40 with mild weakness, myotonia, and cataracts. Lifespan is normal (12).

At present, there is no curative treatment available for DM1. Sudden death can occur, due to myocardial fibrosis and degeneration of the cardiac-conduction system, leading to cardiac arrhytmias (13). There is a high risk of premature death as well, because of restrictions in lung function and alveolar hypoventilation, that could lead to respiratory failure, particularly in the congenital DM1 (9, 13).

The spectrum of sleep disorders that may develop in patients with DM1 includes all types of SDB, secondary causes of insomnia (cramps, myotonia, and pain), restless legs syndrome (RLS), and reduced daytime performance that is independent of nocturnal sleep (14).

EDS is the most frequent non muscular complaint in DM1, affecting approximately 30–39% of patients, with a large patient-reported survey suggesting even higher prevalence rates (87.9%) (15). EDS is among the earliest symptoms patients recall and may even predominate muscular complaints in early disease stages (16).

Bonanni et al. (14) performed a polysomnographic (PSG) study that revealed a prominent dysregulation of REM sleep in DM1, suggesting a possible narcoleptic-like phenotype and alterations in NREM sleep, such as increased sleep instability and impaired delta power dissipation, that contributes to daytime sleepiness. A mean sleep latency <8 min and two or more sleep-onset REM sleep periods (SOREM) in the Multiple Sleep Latency Test (MSLT) were found in four patients (50%), suggesting pathological sleepiness. Five patients showed at least one SOREM (two patients for just one nap, three patients on two naps) and, when including also nocturnal PSG, all these patients had at least two SOREM. Sleep latencies across MSLT naps showed a peculiar trend in DM1, presenting shortest latencies during the first and second nap, whereas the progressively longer latencies on the other naps seem to parallel a progressive “satisfaction” of sleep pressure (14).

Occasionally, in another study patients with DM1 and daytime sleepiness had reduction of cerebrospinal fluid hypocretin levels, typical of patients with Narcolepsy type 1, although they did not carry the HLA-DQB1*06:02 allele (17).

SDB and respiratory dysfunctions are highly prevalent but their direct correlation with EDS remains complex and inconclusive (14). The proportion of patients with sleep apnea was variable from 28 to 93% (16). Depending on the initial results of sleep studies, patients with SDB should be treated using continuous or bi-level Positive Airway Pressure (PAP); for patients requiring Non-Invasive Ventilation (NIV), proving direct treatment benefits can be challenging, as adherence to NIV is suboptimal in a significant number of DM1 patients (18). This may be considered a unique aspect of DM1, adherence might be hindered by a reduced perception of respiratory symptoms or a general apathy, which could potentially be related to cognitive impairment in a subset of patients (19).

Another issue affecting DM patients is orofacial growth impairment early in life (20). Craniofacial muscle weakness can detrimentally impact bone growth during development, particularly in orofacial muscles involved in stimulating growth areas like the intermaxillary synchondrosis, which typically becomes inactive around the age of 15. Due to muscular weakness, craniofacial structures in MD patients may not develop as they should. These subjects may experience a more vertical facial growth than typical subjects, resulting in relatively narrower maxillary arches and narrow palates measured between the palatal shelves, with greater depths (20). These craniofacial changes may contribute to the development of obstructive sleep apnea and EDS by restricting the size of the upper airway, causing upper airway collapse during sleep.

Another SBD to be considered besides OSAS are central sleep apneas and central hypoventilation. Respiratory muscle impairment affects especially REM sleep and leads to hypercapnia which could elicit arousals, resulting in a highly fragmented sleep as well as in sleep hypoventilation, in case of sustained high levels of CO2 (21). At the same time, muscle weakness can result in an ineffective response to high CO2 levels, altering the central ventilation control and posing a risk for central sleep apneas, as well (22).

However, the emergence of SDB abnormalities in DM cannot be solely attributed to muscle weakness. Comparing patients with DM to those with nonmyotonic respiratory muscle weakness, individuals with DM experience higher frequencies of hypoventilation and apneas (central and obstructive) even when they exhibit similar degrees of muscle weakness (measured by maximal inspiratory and expiratory pressures) (23). This suggests that changes in the central nervous system’s control over respiration contribute to abnormal breathing in DM. As a matter of fact, reduced ventilatory responses to hypoxia and hypercapnia, coupled with heightened sensitivity to sedative drugs, indicate a central origin of the breathing impairments in DM (24, 25).

Central nervous system abnormalities occur in both DM type 1 and 2, albeit more severely in type 1. For instance, neurodegeneration in the dorsomedial nuclei of the thalamus can lead to a medial thalamic syndrome characterized by apathy, memory loss, and mental deterioration. Loss of 5-hydroxytryptamine (serotonin) neurons in the dorsal raphe nucleus and the superior central nucleus, along with dysfunction of the hypothalamic hypocretin-orexin system, can lead to short sleep latencies and sleep-onset REM periods during the Multiple Sleep Latency Test (26, 27).

Cortical magnetic stimulation, in combination with phrenic nerve recordings, can be employed to test the corticospinal tract to phrenic motor neuron pathways, offering a reliable method for diagnosing and monitoring patients with impaired central respiratory drive (28). The use of transcortical and cervical magnetic stimulation reveals that over 20% of DM patients exhibit impaired central respiratory drive (29). The presence of neuronal loss in the dorsal central, ventral central, and subtrigeminal medullary nuclei in DM patients with alveolar hypoventilation, along with severe neuronal loss and gliosis in the medullary reticular formation, further supports the presence of central abnormalities (30, 31).

Central sleep dysregulation is also supported by recent neuroradiological findings showing that in DM1 (i) percentage of REM sleep was inversely associated with cerebral grey matter volume; (ii) stage N1 sleep was positively associated with occipital lobe volume; (iii) and stage N2 sleep was associated with amygdala volume (32). Hippocampus volume was positively correlated with self-reported fatigue and somnolence. Linear relationships were also observed between measures of sleep architecture and cognitive performance (32). These findings broadly support the hypothesis that changes in sleep architecture and excessive somnolence in DM1 reflect a primary disease process occurring in the central nervous system (32).

Other factors have been proposed to explain EDS, including dysregulation of the sleep–wake circadian rhythm as neuroendocrine studies showed that the pattern of secretion of growth hormone, prolactin, insulin, cortisol and cytokines had a circadian pathological change in patient with DM1 (33, 34). That is why Liguori et al. (35) considered nocturnal actigraphy data and showed that DM1 patients have a longer time in bed, longer sleep period time, longer actual sleep time and longer sleep latency, a lower degree of regularity in the activity-rest pattern compared to controls. Central phase measurement (the midpoint between ‘Fell asleep’ and ‘Wake up’, expressed as the number of minutes past midnight) was significantly longer in DM1 patients than controls, showing a delayed sleep–wake cycle. Moreover, DM1 patients showed reduced motor activity during daytime and a lower synchronization of the rest-activity rhythm than controls. This study documented that patient with DM1 not only present an impairment of nocturnal sleep, but also show a dysregulation of the sleep–wake circadian rhythm that could enhance EDS; moreover, a reduced amplitude of the circadian rhythmicity was also evident in comparison to controls, probably in relation to the reduced diurnal motor activity of patients due to muscle weakness but also to apathy and cognitive disorder (35).

## Sleep disorders as clinical manifestation of DM2: focus on EDS

Individuals with DM2 exhibit an analogous core symptomatology to those with DM1; however, they also present distinct clinical features (12, 36). Firstly, DM2 typically manifests in adulthood, and it is not associated with a congenital form or a reduced life expectancy, unlike DM1. Lower-limb weakness is the prevailing symptom, impacting the hip flexors and knee extensors muscles, and tendon reflexes are not absent as it commonly occurs in DM1. Other characteristics like muscle atrophy, distal weakness, clinical myotonia, or muscle discomfort may be absent, with muscle pain occasionally taking precedence as the primary complaint. Facial weakness is nearly non-existent. DM2 also encompasses multi-organ involvement, mirroring what is observed in DM1. Despite sharing the same pathological mechanisms with DM1, these differences can be just as severe and should not be underestimated (12, 36).

Additionally, cognitive and behavioral impairments may lead patients to refrain from seeking medical attention (37). Consequently, identifying DM2 can be affected by a diagnostic delay. The actual prevalence of DM2 varies considerably across different regions, and it is estimated to occur at approximately 10% of the rate of DM1 in Southern European Country (DM2 0.99 per 100,000) (38).

While SDB and RLS are the most common sleep disorders observed in DM2, EDS is also highly prevalent impacting approximately 27% (5) of DM2 patients. However, the relationship between EDS and SDB is inconsistent (5). The prevalence of SDB, considering an apnea-hypopnea index above 5 varies from 37.5 to 66.7% while the prevalence of moderate–severe SDB (that is, an apnea-hypopnea index above 15), varies from 0 to 50% (5). The pattern of SDB differs in DM2 as compared with DM1, since respiratory events are mostly obstructive, with a very few observations of central apnea pattern or a mixed (central and obstructive) one.

Surely, a respiratory evaluation should be recommended if obstructive sleep apneas or respiratory muscle dysfunctions are suspected. However, data regarding pulmonary restriction, hypoventilation, and NIV in DM2 patients with EDS are limited. As in DM1, an alteration of REM sleep has been documented also in DM2 by the group of Romigi et al. (4) in which in a polysomnographic study showed that 6 out of 12 patients with DM2 presented REM sleep without atonia (RSWA) and four of them had mild or moderate OSAS. They hypothesized that RSWA, an atypical presence of muscle tone during REM sleep, may represent a compensatory mechanism against nocturnal respiratory events and the brainstem and diencephalon involvement (i.e., pedunculopontine and laterodorsal tegmental nuclei) may activate behavioral states during REM sleep (39, 40).

Fatigue and pain are symptoms that are often reported by patients with DM 2 when discussing sleep disorders. Fatigue may be referred to as EDS, and it is estimated that up to 47.8% of DM2 patients experience fatigue (41). In addition, pain is reported in about 65% of DM2 patients, primarily associated with exercise or cold temperatures, and it negatively affects their quality of life (41). The pathophysiology of pain in DM2 is poorly understood [even if peripheral and central sensitizations are likely to play a prominent role in chronic musculoskeletal pain (42)] and the association between sleep and pain in DM2 patients is still unclear. Tieleman et al. (43) hypothesized a relevant effect of myalgia on nocturnal sleep disruption as 69% of DM2 patients reported pain as the primary cause of sleep impairment (compared with 34% of DM1 patients and 17% of controls) in presence of a very low prevalence of EDS (6.9%) as evaluated by subjective scales (43). Romigi et al. failed to find significant polysomnographic features of sleep impairment in DM patients reporting pain although 58% of subjects reported pain related to sleep disorders through subjective scale (Pittsburgh Sleep Quality Index) (44). Finally, Lam and colleagues (45) found a high prevalence of RLS and EDS in DM2, unrelated to pain or fatigue, in a survey-based study. Whit this evidence, we can hypothesize that despite a crucial role of pain in quality of life in DM2 subjects, its impact on sleep impairment or EDS is not linear.

## Assessment and treatment of EDS in DM

Assessment of EDS is not yet fully elucidated and no specific guidelines or recommendation for EDS in DM are approved (16). Patients with DM frequently report persistent sleepiness that remains unaffected by naps, and this symptom can be highly debilitating, often hindering productive employment. Furthermore, the symptoms of sleepiness overlap with features like mental clouding, fatigue, apathy, and other poorly defined changes in these patients (16).

Laberge et al. (46) delved into the psychometric properties of a five-item Daytime Sleepiness Scale (DSS), incorporating elements derived from the Stanford Sleep Questionnaire and Assessment of Wakefulness in a substantial cohort of 157 patients. Their findings revealed that DSS scores were significantly higher in DM1 patients compared to controls, and this was correlated with muscle weakness. On the whole, DM patients displayed reduced alertness after sleep, a diminished ability to stay awake after a meal, and less restorative sleep. Additionally, fatigue scores were notably elevated in DM1 patients. Conversely, Romigi et al. (47) stated that the DSS score exhibited superior psychometric properties compared to the Epworth Sleepiness Scale (ESS) and should be used in conjunction with the Fatigue and Daytime Sleepiness Scale (FDSS).

Hermans et al. (48) constructed a combined scale for fatigue and daytime sleepiness using Rasch analysis. The resultant scale amalgamates sleep and fatigue items, offering interval measures on a single continuum with minimal floor and ceiling effects. However, this scale has yet to be widely experienced on a large scale.

Besides subjective measures, actigraphic monitoring and PSG with MSLT can be valuable in further characterizing EDS and REM sleep abnormalities.

To exclude OSAS in DM, a polysomnographic is indicated by the American Academy of Sleep Medicine guidelines (49) as the presence of a neuromuscular condition such as DM calls for an exclusion of a concomitant hypoventilation. At the same time, AASM guidelines for MSLT provide the possibility of its usage for residual EDS in OSAS. As for the SBD unrelated cases of EDS, the instrumental diagnostic workups are not specific for DM, but are aligned to the differential diagnosis in cases of somnolence in sleep medicine. According to the symptoms and the information acquired via the questionnaires, the clinical shall orients towards excluding, for example, circadian rhythm disorders via or disorders or central disorders of hypersomnolence via PSG and MSLT, according to the ICSD 3-TR.

Nonetheless, it is essential to note that a MSLT may not comprehensively capture the features of sleepiness reported by patients. Conversely, some individuals with DM1 may exhibit pathological MSLT values without displaying any symptoms of EDS (50).

It is imperative to conduct a comprehensive diagnostic evaluation in neurological patients with EDS. EDS can exacerbate various aspects, including psychiatric symptoms, cognitive deficits, and, in some cases, the severity of the underlying neurological disease itself. Moreover, the quality of life and the risk of accidents are closely linked to EDS. When addressing EDS and fatigue in neurological diseases, it is advisable to pay close attention to lifestyle and sleep hygiene. An individualized approach to managing this symptom in neurological patients should be considered, focusing on modifiable factors such as SDB, psychiatric comorbidities, and medication (15).

DM patients with EDS should be also asked of respiratory involvement symptoms and respiratory function tests including forced vital capacity (FVC), FEV1, oximetry, Maximal Inspiratory Pressure (MIP) and Peak Expiratory Cough Flow (PECF) should be performed as stated by the recent 207th ENMC Workshop on chronic respiratory insufficiency in myotonic dystrophies (21). It was agreed to reserve CPAP in DM if there is only the obstructive component, there are no signs of alveolar hypoventilation and if close follow-up is guaranteed or for those patients in whom BiLevel is not tolerated (for e.g. after 4 weeks). Generally NIV should be commenced when there are the daytime or nighttime symptoms suggestive of chronic respiratory insufficiency in combination with:—Daytime hypercapnia (PaCO2 ≥ 45 mmHg (6.0 kPa) or—FVC < 50% of predicted based on the best of 3 measures and MIP < 60 cmH2O) or evidence of nocturnal hypoventilation (such as: (A) A rise in PaCO2 of ≥8 mmHg (1 kPa) between evening and morning ABGs or other accurate CO2 surrogate. (B) A rise in TcCO2 or ETCO2 > 50 mmHg (6.7 kPa) for more than 50% of total sleep time. (C) While not ideal—when a measure of CO2 is not available—nocturnal oximetry demonstrating sustained oxygen desaturation (SpO2) ≤ 88% for 5 consecutive minutes or SpO2 < 90% for >10% of total sleep time) (21).

Regarding drug interventions, the American Academy of Sleep Medicine guidelines of 2021 (51) suggests that clinicians may consider using modafinil, a wakefulness-promoting medication, for the treatment of hypersomnia secondary to DM in adults. This treatment can be combined with Mexiletine, a class 1B antiarrhythmic agent with a strong affinity for muscle sodium channels. Mexiletine (52) has shown promise in managing excessive daytime sleepiness and reducing myotonia without significant cardiac conduction effects. It is to be noted that despite the AASM conditional indication for the use of modafinil in EDS in DM; prescription in Europe is off-label for DM1 and DM2, although mentioned in the ENMC document of 2014 (21).

Additionally, cognitive-behavioral treatment (CBT) and physical therapy, including exercise programs such as a hand-training program that enhances wrist flexor strength, have demonstrated potential benefits but no specific data on CBT and sleep disorder in DM are present currently in literature. Moreover, neuromuscular electrical stimulations have shown promise in enhancing muscle function and overall well-being for DM1 patients (53, 54).

## Conclusion

In conclusion, this review has provided an in-depth exploration of the prevalence and significance of EDS in the context of DM. EDS impacts on the quality of life of these patients, even in children and adolescents (55), that may have a deleterious impact on work, domestic responsibilities, social life, and a higher risk of accidents (56). An ideal diagnostic approach would include more than one questionnaire (not only ESS but also DSS and FDSS), and instrumental evaluation of EDS should not be reduced to PSG with MSLT but include actigraphy to rule out any dysregulation of the sleep–wake circadian rhythm. While the exact mechanism of EDS in DM remain complex, available evidence supports a central origin, involving two neurotransmitter systems: serotonin and orexin. These neurotransmitter dysregulations can influence respiratory drive contributing to SDB, which often coexists with EDS, but not necessarily.

Further research is needed to elucidate the intricate relationship between EDS and sleep-disordered breathing in these conditions and to develop comprehensive therapeutic strategies to address the clinical burden of EDS in myotonic dystrophy. Finally, this review emphasized a comprehensive, multidisciplinary approach including medical, cognitive-behavioral, and physical therapies to DM1 and DM2 management. It is important to remark that these interventions are vital, given the complex and multisystemic nature of these two syndromes.

## Author contributions

DH: Writing – original draft, Writing – review & editing. AP: Writing – original draft, Writing – review & editing. MM: Writing – review & editing. GR: Data curation, Writing – review & editing, Investigation. MF: Data curation, Writing – review & editing. FT: Data curation, Writing – review & editing, Software. GS: Writing – review & editing, Conceptualization, Supervision. EB: Conceptualization, Supervision, Writing – review & editing.
